# Ethyl pyruvate is a novel anti-inflammatory agent to treat multiple inflammatory organ injuries

**DOI:** 10.1186/s12950-016-0144-1

**Published:** 2016-12-03

**Authors:** Runkuan Yang, Shengtao Zhu, Tor Inge Tonnessen

**Affiliations:** 1 Department of Intensive Care Medicine, Tampere University Hospital, University of Tampere, 10 Bio katu, Tampere, 33014 Finland; 2 Department of Critical Care Medicine, University of Pittsburgh Medical School, 3550 Terrace Street, Pittsburgh, PA 15261 USA; 3 Department of Emergencies and Critical Care, Rikshospital of Oslo University, PO Box 4950, Nydalen, Oslo 0424 Norway; 4 Department of Gastroenterology, Beijing Friendship Hospital, Capital Medical University, 95 Yong An Road, Beijing, 100050 China; 5 Institute for Clinical Medicine, University of Oslo, Blindern, Oslo 0316 Norway

**Keywords:** Ethyl pyruvate, Inflammation, Reactive oxygen species, HMGB1

## Abstract

Ethyl pyruvate (EP) is a simple derivative of pyruvic acid, which is an important endogenous metabolite that can scavenge reactive oxygen species (ROS). Treatment with EP is able to ameliorate systemic inflammation and multiple organ dysfunctions in multiple animal models, such as acute pancreatitis, alcoholic liver injury, acute respiratory distress syndrome (ARDS), acute viral myocarditis, acute kidney injury and sepsis. Recent studies have demonstrated that prolonged treatment with EP can ameliorate experimental ulcerative colitis and slow multiple tumor growth. It has become evident that EP has pharmacological anti-inflammatory effect to inhibit multiple early inflammatory cytokines and the late inflammatory cytokine HMGB1 release, and the anti-tumor activity is likely associated with its anti-inflammatory effect. EP has been tested in human volunteers and in a clinical trial of patients undergoing cardiac surgery in USA and shown to be safe at clinical relevant doses, even though EP fails to improve outcome of the heart surgery, EP is still a promising agent to treat patients with multiple inflammatory organ injuries and the other clinical trials are on the way. This review focuses on how EP is able to ameliorate multiple organ injuries and summarize recently published EP investigations.

**Graphical Abstract:**
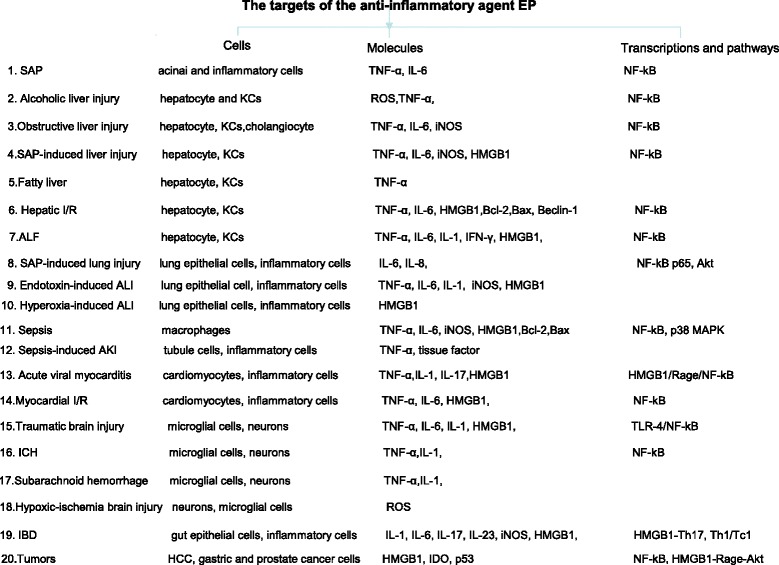
The targets of the anti-inflammatory agent EP

The targets of the anti-inflammatory agent EP

## Background

Pyruvate is the final product of glycolysis and the starting substrate for the tricarboxylic acid (TCA) cycle, and this important metabolic intermediate is also an effective scavenger of hydrogen peroxide and other ROS [1, 2]. Pharmacological administration of pyruvate is able to improve organ function in animal models of oxidant-mediated cellular injury [1, 2]; however, pyruvate is unstable in aqueous solutions and this certainly limits its therapeutic potential. EP, a simple derivative of pyruvic acid, is also an ROS scavenger, but exerts pharmacological effects, such as the anti-inflammatory effects, which are quite distinct from those exerted by pyruvate anion [1, 2]. Treatment with EP has been shown to improve survival and/or ameliorate multiple organ dysfunctions in a wide variety of preclinical models of critical illnesses [1, 2]. Up to date, about 340 EP related papers have been published; about 100 new papers published after 2010 have not been summarized and reviewed. This review focuses on how EP is able to ameliorate inflammatory injures of multiple vital organs and summarizes new findings from recently published EP investigations.

## EP ameliorates severe acute pancreatitis (SAP) and attenuates SAP related distant organ injury

Acute pancreatitis (AP) is a relatively common disease, its severe form is potentially fatal and SAP is associated with high mortality, ranging from 15–40% [3–8]. The inflammatory cytokines play a crucial role in the pathogenesis of SAP [3, 8, 9]; furthermore, the damaged pancreatic acinar cells and the activated inflammatory cells produce a large amount of oxygen radicals in AP, and these ROS molecules can damage the lipid membranes of pancreatic acinar cells, they can also injure the capillary endothelium in the circulation to accelerate the progress of SAP [7]. Currently, therapeutic efforts are limited to supportive measures, because no effective specific treatment exists.

### The effect of EP on acute pancreatitis

EP has been repeatedly reported to ameliorate SAP in different animal models [4, 6, 10, 11]. EP treatment [EP source in this review was all from Sigma-Aldrich unless otherwise noted. EP dissolved in commercially available Ringer’s lactate solution (RLS). Commercially available RLS was used as the control solution. EP (40 mg/kg) was intraperitoneally injected every 6 h for 48 h)] significantly ameliorates pancreatic injury and necrosis [4, 6]; EP therapy also markedly reduces pancreatic expression of TNF-α, IL-6, HMGB1 and NF-kB DNA binding [4, 6]; treatment with EP reduces the number of inflammatory cell infiltration and decreases the pancreatic level of lipid peroxidation, which is a parameter of ROS [4]. Because early inflammatory cytokines (such as TNF-α and IL-6), late inflammatory mediator HMGB1 and ROS play a significant role in the pathogenesis of SAP [4, 6, 10, 11], and EP can reduce the levels of these inflammatory cytokines and scavenge ROS. Therefore, EP may attenuate pancreatic injury during SAP.

### The effect of EP on severe acute pancreatitis related multiple organ injuries

About 20–30% of all acute pancreatitis patients develop SAP in clinical practice, and the mortality rate in SAP is 20–30% [3]. SAP starts as a local inflammation of pancreatic tissue that induces the development of multiple extrapancreatic organs dysfunction [6, 8]. During SAP, the concentrations of both early (TNF-α, IL-6) and late inflammatory cytokines are significantly increased [6, 10–13], these cytokines play a significant role in the pathogenesis of SAP [6, 12]. The late inflammatory cytokine HMGB1 is particularly important because extracellular HMGB1 can aggravate the pancreatic inflammatory process [14] and HMGB1 can also contribute to multiple distant organ injuries in the following experimental models as well: HMGB1 contributes to liver injury in ischemia-reperfusion [15]. Exogenous HMGB1 injection is able to induce liver injury in normal mice [16]. HMGB1 impairs hepatocyte regeneration during acetaminophen hepatotoxicity and blockade of HMGB1 improves hepatocyte regeneration in acetaminophen overdose-induced fatal liver injury [17]. Anti-HMGB1 treatment protects against APAP hepatotoxicity in rats [18]. HMGB1 also contributes to renal ischemia reperfusion injury [19], sepsis-induced kidney injury [20] and severe acute pancreatitis related kidney injury [21]. HMGB1 is also found to significantly contribute to hemorrhagic shock related acute lung injury (ALI) [22]; hypoxia-induced ALI [23] and severe acute pancreatitis related ALI [24]. HMGB1 is also an important factor that not only significantly contributes to gut mucosal injury [16], but also mediates gut bacterial translocation (BT) [25], and blockade of HMGB1 can even prevent gut BT [25]. Gut mucosal injury and intestinal BT in SAP is particularly important because the intestine is the biggest reservoir of bacteria in the body and leakage of bacteria or microbial products, notably LPS, from the lumen of the gut into the systemic circulation, which drives the development of systemic inflammation and multiple organ dysfunction syndrome (MODS) in experimental models [26]. SAP frequently induces gut barrier dysfunction [6, 9, 27, 28]. The small intestine becomes damaged by intestinal ischemia-reperfusion during SAP [29, 30], and the failure of gut barrier is associated with BT [29], which is evident in SAP [6, 27, 28, 31]. SAP patients have significantly increased serum LPS [32], 68.8% of the SAP patients have bacteraemia and these bacteria are highly likely gut-derived opportunistic pathogens [27]. Furthermore, BT and infected pancreatic necrosis in acute necrotizing pancreatitis derive from small bowel rather than from the colon [28]. BT and/or gut derived LPS play a critical role in the development of systemic inflammatory response syndrome (SIRS) and MODS during SAP [29, 33], because BT not only contributes to pancreatic infection [28, 33], but also induces/triggers SIRS/sepsis in critical illness [33, 34]. The profound SIRS/sepsis can lead to MODS and mortality in SAP [9, 29, 31, 33], this is one of the underlying mechanisms that AP frequently affects extrapancreatic organs [6, 25], and the incidence of MODS in SAP is not available but certainly higher than the 20–30% of mortality rate in SAP [3]. Systemic inflammation with multi-organ dysfunction is the cause of death in a murine ligation-induced SAP, and SIRS and MODS can lead to the preponderance of mortality (75%) in this lethal SAP model [9] while bile duct ligation does not have mortality, even though obstructive jaundice is prone to sepsis [9]. Therefore, the gut is thought to act as the starter of SIRS [29] and HMGB1 may be an important factor that links gut barrier dysfunction and MODS during SAP. EP (40 mg/kg was intraperitoneally injected every 6 h for 48 h) can ameliorate SAP related multiple organ injuries [6, 10, 11] at least partly because EP not only reduces the inflammation in these organs [6, 10, 11], but also inhibits nearly 90% of the hepatic tissue HMGB1 to release [12] and other related cells to release HMGB1 [10, 12], thereby decreases the circulating HMGB1 level in SAP [10, 14]. Thus, HMGB1 is an important factor that not only directly contributes to multiple organ injuries, but also mediates gut BT to trigger/induce systemic inflammation/sepsis, the latter can lead to MODS. Currently, the circulating HMGB1 level is thought to be reliable to predict the severity of SAP [10, 14], this is likely because HMGB1 is linked to multiple organ injuries during SAP [10, 12, 14], the liver is an important source of circulating HMGB1 [12], and the circulating HMGB1 level could reflect the severity of liver injury, which is one of the important parameters for diagnosing SAP [6, 12]. SAP associated BT was reduced by 90% following EP treatment [6] breaking the link between BT and MODS. Therefore, EP could be a better treatment option against SAP related multiple organ injuries in experimental models [6, 10, 11].

### The effect of EP on liver injuries

The liver is the largest organ in the body, hepatocytes account for 70–80% of the hepatic cytoplasmic mass and non-parenchymal cells make up 20–30% of the hepatic cytoplasmic mass [35]. Kupffer cells (KCs) are the most abundant mononuclear phagocytes in the body and a predominant source of inflammatory cytokines released into the systemic circulation [8]. The amount of cytokines released from the liver represents about 50% of the total cytokine content in the body [36], suggesting that the liver is the major contributor of the circulating cytokines.

### EP ameliorates experimental alcoholic liver injury

Alcoholic hepatitis is associated with considerable morbidity; 46% of patients with the severe alcoholic hepatitis die within 30 days of the onset [37]. General measures for treatment include abstinence from alcohol and supportive care. Alcoholic hepatitis is reversible if the patient stops drinking, it usually takes several months to resolve, however, abstinence from alcohol is difficult, and the treatment is still challenging. In an experimental murine model, alcohol induces fatty change and piecemeal necrosis; alcohol administration also induces significantly increased hepatic lipid peroxidation, NF-kB activation, TNF-α mRNA expression; furthermore, alcohol administration induces a large amount of gut BT, which can serve as the “second hit” to contribute to the alcoholic liver injury. All of these alcohol-induced effects are ameliorated by treatment with EP (40 mg/kg was intraperitoneally injected every 6 h for 48 h) instead of Ringers lactate solution, suggesting that EP ameliorates hepatic inflammatory response and hepatic lipid peroxidation, and resultantly decreases hepatocellular injury duo to acute alcoholic intoxication [37].

### EP ameliorates experimental obstructive jaundice induced liver injury

Obstructive jaundice and cholangitis are common diseases that are prone to sepsis that can lead to mortality [38]. In an experimental murine model of common bile duct ligation model [38], obstructive jaundice induces evident hepatocellular necrosis and significantly increased circulating ALT and total bilirubin levels. Obstructive jaundice also induces increased hepatic lipid peroxidation and increased hepatic expression of transcripts for TNF-α, IL-6, and iNOS. Furthermore, bile duct ligation also induces a large amount of gut BT, which can serve as the “second hit” to contribute to the liver injury. All of these changes can be significantly attenuated by delayed treatment with EP (40 mg/kg was intraperitoneally injected every 8 h for 72 h) instead of RLS, suggesting that EP ameliorates hepatic inflammation, lipid peroxidation and necrosis in obstructive jaundice [38]. In addition, EP treatment increases NF-kB DNA binding, which often modulates inflammation when hepatic necrosis is not evident [37] but modulates regeneration when hepatic necrosis is evident [38], this concept has been proved in acetaminophen hepatotoxicity in which massive hepatocyte necrosis is a predominant feature [39, 40]. Therefore, it is likely that EP enhances NF-kB DNA binding to improve hepatocyte regeneration in obstructive jaundice.

### EP ameliorates acute liver injury secondary to severe acute pancreatitis

SAP frequently affects the liver and the inflamed liver play a significant role in the pathogenesis of SAP [6, 12, 13]. In a lethal experimental SAP murine model, SAP induces significantly increased hepatic lipid peroxidation, NF-kB activation, hepatic expression of transcripts for TNF-α, IL-6, iNOS and COX-2 [12]. SAP also induces focal hepatocyte necrosis and a large number of inflammatory cell infiltration [6, 12, 13], all of these changes can be significantly decreased by delayed treatment with EP instead of Ringers lactate solution [12]. In particular, SAP can induce the loss of nearly 85% of hepatic tissue HMGB1 tested by western blot using whole hepatic tissue, and this effect can be reversed by EP treatment (40 mg/kg intraperitoneally injected every 6 h for 48 h) [12], suggesting that the liver is an important resource of the circulating HMGB1, and EP is a potent HMGB1 inhibitor [12]. In another SAP rat model, SAP induces significantly increased hepatic lipid peroxidation, hepatic NF-kB DNA binding and hepatic expression of transcripts for TNF-α, IL-1 and HMGB1 [13], all of these changes can be significantly reduced by EP treatment (40 mg/kg intraperitoneally injected every 6 h for 48 h) [13].

### The effect of EP on fatty liver

Fatty liver is common world widely, and its treatment is problematic. In a high-fat induced rat model, the fatty liver induces increased serum ALT and increased hepatic TNF-α level; these changes can be significantly decreased by EP intake (supplemented in 0.3% drinking water for 6 weeks) but EP does not affect the development of a fatty liver [41], suggesting that EP can protect against inflammation induced liver cell damage but EP cannot prevent the development of fatty liver in animal experiment.

### The effect of EP on hepatic ischemia-reperfusion injury

Hepatic ischemia-reperfusion (I/R) is a pivotal clinical problem occurring in many clinical conditions such as transplantation, trauma and hemorrhagic shock [42]. Hepatic I/R induces significantly increased hepatic expression of TNF-α, IL-6, HMGB1 and NF-kB activation, and hepatic I/R also induces markedly increased hepatic expression of Bcl-2, Bax, Beclin-1 and LC3, which play an important role in the regulation of intrinsic pathway of apoptosis and autophagy, all of these changes are significantly reduced by EP treatment (1 h before the ischemia procedure, a single dose of EP was intraperitoneally injected to animals in the 20 mg/kg group, the 40 mg/kg group and the 80 mg/kg group, liver tissue samples were obtained 4 h, 6 h and 16 h after I/R), suggesting that EP ameliorates hepatic I/R injury via its anti-inflammatory and its anti-apoptosis effect.

### The effect of EP on acute fatal liver injury

Drug-induced acute fatal liver injury is the leading cause of acute liver failure (ALF) in the developed countries [43–46], and ALF has a high mortality and the treatment is still quite challenging [43].

Concanavalin A induces autoimmune hepatitis with significantly increased hepatic expression of TNF-α, IL-6, IL-1, HMGB1 and NF-kB activation, EP treatment (1 h before the Con A injection, a single dose of EP was intraperitoneally injected to the animals in the 40 mg/kg EP group and the 80 mg/kg EP group; liver tissue samples were obtained 3 h, 6 h and 24 h after Con A injection) reduces all of these changes and resultantly ameliorates Concanavalin induced autoimmune hepatitis [44], which can be fatal.

D-galactosamine induces acute fatal liver injury with significantly increased serum TNF-α, HMGB1, IFN-gamma and endotoxin, all of these changes can be significantly decreased by EP treatment (A single dose of 40 mg/kg EP was intraperitoneally injected 2 h after ALF induction, samples were taken 22 h after EP injection), therefore, EP can ameliorate acute fatal liver injury induced by D-galactosammine [45].

Acute-on-Chronic liver failure (ACLF) rats have significantly increased serum endotoxin, TNF-α, HMGB1, IFN-gamma and IL-18, EP administration (40 mg/kg was intraperitoneally injected at 3 h, 6 h, 12 h, 24 h after the induction of ACLF, and samples were taken 48 h after the induction of ACLF) decreases all of these changes to protect against ACLF in rats [46].

Acetaminophen is the leading cause of drug induced ALF, and EP treatment (40 mg/kg was intraperitoneally injected every 8 h for 24 h) can reduce liver injury at early phase by its potent anti-inflammatory effect [43].

### The effect of EP on diabetes induced liver injury

Diabetes can lead to an increased oxidative stress that significantly contributes to diabetes-induced liver injury [47]. Diabetes induces significantly increased total antioxidant status and hepatic peroxidation. EP therapy (50 mg/kg was intraperitoneally injected twice a day for 14 days) (instead of Ringer solution) significantly decreases all of these changes and resultantly ameliorates diabetes-induced liver injury in a streptozocin induced diabetic rat model [47].

## The effect of EP on acute lung injuries

### EP on SAP related acute lung injury

Acute lung injury (ALI), also addressed as mild acute respiratory distress syndrome (MARDS), is a significant health problem associated with high mortalities [8, 48]; MARDS is also a severe complication and a major feature of MODS to SAP [8]. In patients with AP, up to 20% of all deaths occur prior to admission to hospitals, and MARDS is the predominant cause of death in these cases [48]. In SAP, the MARDS is the main contributing factor to early deaths, i.e. within the first week after admission [49]. The inflammatory mediators and the profound SIRS are thought to play a key role in the development of MARDS [3, 50, 51] because the significantly increased serum inflammatory mediators activate alveolar macrophages to release chemotactic mediators that play an important role in recruiting neutrophils, which work together with the elevated circulating pro-inflammatory mediators to severely damage alveolar epithelium and microvascular endothelium, and resultantly causes the increased permeability of the alveolar-capillary barrier and pulmonary edema. This theory is supported by the following evidence in which the alveolar-capillary barrier is severely injured and the pulmonary permeability is significantly increased in an experimental acute necrotizing pancreatitis [6]. The anti-inflammatory agent EP (40 mg/kg intraperitoneally injected every 6 h for 48 h) markedly decreases the lung permeability and alleviates pulmonary edema at least partly by reducing pulmonary inflammation and neutrophils infiltration in a couple of experimental SAP models with acute lung injury [6, 10, 52, 53]. Increased local and systemic levels of IL-6 are associated with inflammatory process, including neutrophil infiltration of the alveolar space, resulting in lung injury [54]. EP treatment reduces the IL-6-induced release of IL-8 and decreases IL-6-induced neutrophil adhesion to the lung epithelial cells [54], and this anti-inflammatory effect is via Akt and NF-kB p65 pathway [55]. Therefore, EP reduces secretory and adhesive potential of lung epithelial cells under inflammatory conditions [54, 55].

### The effect of EP on endotoxin-induced acute lung injury

LPS intravenous injection induces significantly increased plasma TNF-α, IL-6; LPS administration also induces significantly increased expression of HO-1 and iNOS in lung tissue. In addition, LPS also induces lung edema and infiltration of neutrophils, all of these changes can be reduced by EP treatment (intravenously infused for 4 h into the animals in the following 3 different EP concentration groups: 20, 40 and 60 mg/kg, and lung tissue samples were harvested 6 h after EP administration) [56]. In a murine model, LPS intratracheal administration significantly increases the release of TNF-α, IL-6, IL-1 and HMGB1 into bronchoalveolar lavage, all of these changes can be markedly decreased by EP treatment (100 mg/kg was intraperitoneally injected at 0 h, 12 h, 24 h and 48 h after the induction of ALI), and early administration of EP can improve survival [57].

### The effect of EP on hyperoxia-induced acute lung injury

Prolonged exposure to hyperoxia results in ALI, accompanied by significantly increased levels of proinflammatory cytokines and a large number of leukocyte infiltration in the lungs [58]. HMGB1 plays a critical role in mediating hyperoxia induced ALI through the recruitment of leukocytes into the lung [58], a single dose of EP treatment (40 mg/kg intraperitoneal injection prior to hyperoxia exposure, and lung tissue samples were taken 24 h after hyperoxia exposure) attenuates the hyperoxia induced ALI probably by inhibiting HMGB1 secretion from hyperoxic macrophages [58], suggesting that EP may treat oxidative inflammatory lung injury in patients receiving hyperoxia through mechanical ventilation.

## The effect of EP on macrophages, systemic inflammation and sepsis

### EP inhibits LPS-stimulated macrophages to release both early and late inflammatory cytokines

In cultured macrophages, LPS stimulates the macrophages to release TNF-α, IL-6, and HMGB1, and these changes can be effectively prevented by EP treatment (cells were incubated with 25 mM EP for 48 h) [59]. LPS stimulates macrophages to release HMGB1 and up-regulates iNOS expression, EP treatment (cells were incubated with 25 mM EP for 24 h) can reverse these effects by inducing heme oxygenase-1 (HO-1) via a p38 MAKP- and NRF2-dependent pathway [60]. HMGB1 is a ubiquitous nuclear protein that can be actively secreted by immunocompetent cells, including monocytes, macrophages and neutrophils, and this highly conservative nuclear protein is an important late inflammatory mediator in sepsis [61]. HMGB1 can also be passively released by dying cells or necrotic tissue [25]. HMGB1 plays an important role in modulating inflammatory cascade in activated macrophages: HMGB1 stimulates macrophages to release TNF-α and IL-6, while HMGB1neutralizing antibody can block TNF-α release [62, 63] and knocking-out HMGB1 receptor can reverse IL-6 release [63]. In macrophages cultures, LPS stimulates macrophages to release high concentrations of early inflammatory cytokines such as TNF-α, IL-6 and IL-1 and the late mediator HMGB1 [61–63], and EP treatment reduces these changes by specifically inhibiting the activation of p38 mitogen activated protein kinase and NF-kB, two signalling pathways that are critical for cytokines release [61].

### EP prevents lethality in mice with sepsis and systemic inflammation

Sepsis is a serious clinical syndrome, which is mediated by an early (such as TNF-α and IL-1) and late (such as HMGB1) pro-inflammatory cytokine response to infection [61]. Delayed treatment with EP (40 mg/kg intraperitoneally injected 24 h, 30 h, 48 h, and 54 h after cecal puncture, the experiment finished 120 h after cecal puncture) significantly increases survival and markedly reduces circulating levels of HMGB1 in mice with sepsis [61]. EP increases survival and decreases serum HMGB1 by up-regulation of HO-1 level in septic animals [60]. In an established septic animal model, sepsis induces significantly increased plasma TNF-α, IL-6 and IL-1; sepsis also increases hepatic lactate, lactate/pyruvate levels and down-regulates hepatic ATP and energy charge levels; all of these effects can be reversed in the septic mice treated with a single dose of EP (40 mg/kg intraperitoneal injection, and the liver samples were taken 6 h after EP injection), suggesting that EP protects against sepsis by regulating energy metabolism and inhibiting systemic inflammation [64]. In addition, EP improves sepsis outcome by reducing mitochondrial swelling and dysfunction in experimental sepsis [65]. Septic patients have significantly increased serum HMGB1 levels, which can induce endothelial cell hyperpermeability via BAX and BCL-2 regulated apoptosis, EP can reverse these detrimental effects to prevent endothelial cell injury in experimental sepsis [66]. Furthermore, EP can effectively reduce vascular endothelial inflammation and this effect at least partly depends on the attenuation of endoplasmic reticulum stress [67].

## The effect of EP on acute kidney injuries

Acute kidney injury (AKI) is a common serious complication of SAP and sepsis. Endotoxin and ROS play an important role in the pathogenesis of AKI and SAP.

### The effect of EP on sepsis-induced acute renal failure

Sepsis is a common cause of acute renal failure (ARF), and the incidence of sepsis increases markedly after age of 50 [68]. Sepsis induces significantly increased plasma TNF-α, creatinine and causes tubular damage and multiple organ injury, sepsis also induces increased mRNA for TNF-α, tissue factor, PAI-1, and urokinase-like plasminogen activator; all of these changes can be significantly decreased by EP treatment (a single dose of 40 mg/kg was intraperitoneally injected 12 h after cecal puncture and experiment finished 24 h after cecal puncture), therefore, EP attenuates sepsis-induced ARF in an animal model [68].

### The effect of EP on diabetic nephropathy

Diabetic nephropathy is a common complication [69]. Diabetic rats have increased laminin, type IV collagen and fibronectin deposition in the glomeruli and these animals also have albuminuria and increased NADPH-dependent ROS generation; all of these changes can be significantly decreased by EP treatment (40 mg/kg intraperitoneally injected every other day for 12 weeks) [69], suggesting that EP might be a novel therapy to treat diabetic nephropathy.

### The effect of EP on cisplatin-induced nephrotoxicity

Cisplatin-induced nephrotoxicity is common in clinical practice. Cisplatin induces significantly increased perfusion pressure, serum urea, creatinine, total oxidant status and tissue lipid peroxidation, all of these changes can be significantly decreased by EP therapy (50 mg/kg was intraperitoneally injected once a day for 5 days) [70], suggesting that EP has a protective effect against cisplatin nephrotoxicity.

## The effect of EP on heart injury

### EP attenuates acute viral myocarditis

Inflammation plays important roles in the pathogenesis of coxsackievirus B3 (CVB3)-induced acute viral myocarditis (AVMC) [71]. CVB3 virus induces increased levels of HMGB1, TNF-α, IL-1, IL-17 in the heart and serum of the AVMC mice, and all of these changes can be significantly decreased by EP treatment (40 mg/kg/day and 80 mg/kg/day intraperitoneally injected on day 5, day 6 and day 7 post infection), and this protective effect is associated with inhibition of HMGB1/RAGE/NF-kB pathway [71].

### EP protects against myocardial ischemia/reperfusion

HMGB1 is a late inflammatory cytokine that triggers and amplifies the inflammation cascade following ischemia/reperfusion (I/R), and EP can inhibit HMGB1 release in I/R models [72]. I/R procedure induces increased levels of HMGB1, TNF-α, IL-6, these changes can be significantly reduced by EP treatment (a single dose of EP with 40 mg/kg concentration was intravenously injected prior to the 48 h reperfusion) instead of PBS, the accumulation of HMGB1 is deleterious to the heart following myocardial I/R [72]. In another rat cardiac I/R model, EP treatment significantly preserves cardiac function, enhances tissue ATP levels, attenuates myocardial oxidative injury and reduces apoptosis following myocardial ischemia [73]. In another regional heart I/R rat model, EP therapy (a single dose of 40 mg/kg was intraperitoneally injected 1 h prior to the 24.5 h I/R procedure) inhibits I/R-induced NF-kB translocation and neutrophil activation in myocardium, EP also decreases the serum levels of inflammatory cytokines, and resultantly EP improves cardiac function and reduces infarct size after regional I/R injury [74]. In another prolonged rat myocardial ischemia model, EP therapy enhances myocardial ATP levels, attenuates myocardial oxidative injury, and resultantly decreases infarct size and preserves cardiac function [75].

## The effect of EP on acute brain injury

### EP attenuates traumatic brain injury

In a rat model of unilateral cortical contusion injury (CCI), EP treatment (40 mg/kg was intraperitoneally injected 1 h, 12 h and 24 h after brain injury, brain samples were harvested 72 h after brain injury) significantly decreases the number of dead/dying cells in the ipsilateral hippocampus and improves recovery of beam-walking, neurological scores after injury, suggesting that EP treatment after CCI is neuroprotective and improves neurobehavioral recovery [76]. Traumatic brain injury (TBI) can cause brain cell death/dying, and the/dead/dying cells can release nuclear protein HMGB1 that can activate inflammatory pathways, therefore, the HMGB1 inhibitor EP (75 mg/kg was intraperitoneally injected at 5 min, 1 h, 6 h after brain injury, and brain samples were harvested 24 h after brain injury) significantly decreases the expressions of HMGB1, TLR4, NF-kB DNA binding and inflammatory mediators, such as, TNF-α, IL-1 and IL-6. EP treatment also ameliorates beam walking performance, brain edema and cortical apoptotic cell death, suggesting that the protective effects of EP maybe mediated by the reduction of HMGB1/TLR4/NF-kB-mediated inflammatory response in the injured rat brain [77]. Many TBI survivors sustain neurological disability and cognitive impairment due to the lack of defined therapy to reduce TBI-induced long-term brain damage, EP (40 mg/kg was intraperitoneally injected at 15 min, 12 h, 24 h, 36 h, 48 h, 60 h after brain injury, and brain samples were taken 28 days after brain injury) confers long-term neuroprotection against TBI, possibly via breaking the vicious cycle among matrix metalloproteinase-9-mediated blood–brain barrier disruption, neuroinflammation and long-lasting brain damage [78]. Experimental TBI is known to produce an acute increase in cerebral glucose utilization, followed rapidly by a generalized cerebral metabolic depression. Early administration of EP (40 mg/kg was intraperitoneally injected at 0 h, 1 h, 3 h, 6 h after brain injury, and brain samples were harvested 24 after brain injury) enhances cerebral glucose metabolism and neuronal survival, therefore, EP therapy is able to attenuate cerebral metabolic depression and neuronal loss after traumatic brain injury [79].

### EP ameliorates acute intracerebral haemorrhage-induced brain injury

Intracerebral haemorrhage (ICH) is a devastating disease with no specific treatment. Increasing evidence indicates that inflammatory response plays an important role in ICH-induced brain damage [80, 81]. In a murine model of ICH, EP treatment (3 concentrations of EP at 10 mg/kg, 50 mg/kg and 100 mg/kg were intraperitoneally injected to animals in 3 separate groups at 1 h, 6 h, 12 h after the induction of ICH, and brain samples were harvested 72 h after the induction of ICH) reduces brain edema and improves neurological function after ICH. EP also protects neurons from haemoglobin-induced cell death in vitro and neuronal cell degeneration in ICH mice. EP exerts anti-inflammatory effects via inhibiting microglia activation, NF-kB activation and decreasing TNF-α, IL-1 production. These results indicate that EP protects ICH induced brain damage via anti-cell death and anti-inflammatory actions [80]. In another rat ICH model, EP treatment (40 mg/kg was intraperitoneally injected at 1 h, 6 h and 12 h after the induction of ICH, and brain samples were harvested 72 h after the induction of ICH) significantly reduces inflammatory cell infiltration and expression of IL-1, matrix metalloproteinase-9 in the perihematoma after ICH. EP therapy also shows less brain edema, less haemorrhage and greater neurobehavioral function. The results suggest that EP ameliorates inflammatory damage after ICH via HMGB1-RAGE signalling pathway [81].

### EP alleviates early brain injury induced by subarachnoid hemorrhage

Subarachnoid hemorrhage (SAH) is also a devastating disease with no specific treatment [82]. In a rat model of SAH, EP treatment (A single dose of EP at 100 mg/kg concentration was intraperitoneally injected 1 h after the induction of SAH, and brain samples were harvested 24 h after the induction of SAH) inhibits microglia activation and reduces the expression of inflammatory cytokines TNF-α and IL-1; EP therapy also inhibits apoptosis and prevents the disruption of tight junction proteins to stabilize the BBB [82].

### EP decreases the cerebral ischemic injury

In a rat cerebral ischemia model, EP administration (intraperitoneally injected at the doses of 1, 4, 20 and 40 mg/kg at 4 h and 24 h after the brain ischemia injury, and the size of infarct was assessed after 2 days of reperfusion) significantly reduces infarct volume and also suppresses the infarct volume related motor impairment, neurological deficits, microglial activation and inflammatory cytokine expression. Furthermore, the neuroprotective effect is still evident even when the EP treatment is given as late as 24 h after the cerebral ischemia induction, suggesting that EP can protect against cerebral ischemia injury with a wide therapeutic window [83].

### EP exerts neuroprotective effects against hypoxic-ischemic brain jury

Neonatal hypoxic-ischemic (HI) brain injury causes severe brain damage in newborns. Following HI injury, rapidly accumulating oxidants injure neurons and interrupt ongoing developmental processes [84]. EP therapy (a single dose of EP at 25 mg/kg was intraperitoneally injected 30 min after HI brain injury, and brain samples were harvested at 3 h, 6 h, 12 h, 24 h,48 h,72 h, 7 days and 4 weeks after HI brain injury) and the insulin-like growth factor-1 (IGF-1) treatment protect the neonatal rats brain against HI injury and improve neurological performance and these effects are additive [84].

## The effect of EP on inflammatory bowel disease

Inflammatory bowel disease is characterized by overproduction of inflammatory mediators and reactive oxygen that induce intestinal damage and chronic inflammation. Inflammatory bowel disease is common but the treatment is still challenging [85, 86]. In a rat TNBS-induced colitis model, EP treatment (20 mg/kg, 40 mg/kg and 100 mg/kg were orally administered to 3 separate groups once a day for 7 days) significantly recovers the mucosal cytoarchitecture by reducing neutrophil infiltration and decreasing the levels of multiple inflammatory mediators (IL-1, IL-17, IL-6, IL-23, iNOS) [86]. EP therapy (40 mg/kg was intraperitoneally injected once a day for 7 days) also ameliorates experimental colitis in mice by inhibiting the HMGB1-Th17 and Th1/Tcl responses [85].

## The effect of EP on tumour

As inflammation is linked to cancer growth, the anti-inflammatory agent EP is expected to have anti-tumor activity, and EP administration (40 mg/kg and 80 mg/kg intraperitoneally injected once a day for 9 days) significantly inhibits hepatic tumor growth [87]. The low-cost EP (40 mg/kg intraperitoneally injected twice a day for 3 weeks) elicits a potent immune-based antitumor response through inhibition of indoleamine 2, 3-dioxygenase (IDO), a key tolerogenic enzyme for many human tumors [88]. EP treatment (40 mg/kg intraperitoneally injected once a day for 2 weeks) inhibits tumor angiogenesis by inhibition of the NF-kB signalling pathway [89]. HMGB1 and RAGE are significantly expressed in gastric adenocarcinoma, and EP treatment (40 mg/kg and 80 mg/kg intraperitoneally injected once a day for 2 weeks) inhibits gastric cancer growth via regulation of the HMGB1-RAGE and Akt pathways [90]. EP administration (cultured cancer cells were treated with EP at 10 mM and 20 mM for up to 120 h) also inhibits growth and invasion of gallbladder cancer cells via down-regulation of the HMGB1-RAGE axis [91]. Hepatocellular carcinoma (HCC) develops in response to chronic hepatic injury. Although p53 is usually regarded as a tumor suppressor, its constant activation can promote pro-tumorigenic inflammation, at least in part, via inducing HMGB1 release. EP administration (40 mg/kg was intraperitoneally injected once every other day for 7 weeks) prevents tumorigenesis in rat livers by restoring p53, and EP treatment does not affect p53-mediated hepatic apoptosis [92]. Furthermore, EP treatment (Cultured cancer cells were treated with 10 mM, 20 mM EP up to 72 h) induces apoptosis and cell-cycle arrest in G phase in hepatocellular carcinoma cells [93]. In addition, EP treatment (Cultured cancer cells were treated with 10 mM, 20 mM, 40 mM EP up to 120 h) defangs some malignancy-associated properties of prostate cancer cells including proliferation, invasion and anchorage-independent growth [94]. Taken together, EP may have a potential as a new multi-functional compound for cancer therapy.

## Molecular mechanisms responsible for the anti-inflammatory effects of EP

EP does not inhibit nuclear translocation of NF-kB family members but attenuates NF-kB DNA binding in an experimental colitis model [95], more specifically, EP inhibits NF-kB activation by alkylating a critical cysteine residue (Cys^38^) in the p65 subunit of the NF-kB heterodimer, and alkylation of Cys^38^ interferes with DNA-binding by the transcription factor [1, 2]. EP also interacts with NF-kB subunits, Rel A and p50 to inhibit their functions at multiple points, for example, EP is able to inhibit the nuclear association of Rel A after TNF-α treatment [96]. At least some of the anti-inflammatory effects EP are related to its ability to scavenge ROS, since EP is an anti-oxidant [1, 2, 12], and oxidative stress is able to activate NF-kB-dependent gene transcription [1, 2, 12]. EP not only prevents nuclear-to-cytoplasmic translocation of HMGB1 [95], but also inhibits cytoplasmic HMGB1 to be released extracellularly [12]. Moreover, EP inhibits HMGB1 release from primary microglial cells via direct intracellular Ca (2+) chelation [97], and EP also regulates inflammation and exerts a neuroprotective effect via dual functions, chelating intracellular Zn (2+) and promoting NAD replenishment [98].

## EP administration on translational/clinical practice

EP therapy has been confirmed to be effective and safe in multiple SAP animal models and multiple liver injury models, it would be reasonable to focus on SAP and alcoholic hepatitis clinical trials next step. Intravenous infusion is used to administer EP (from Critical Therapeutics Inc, Lexington, MA, USA) to healthy volunteers and high-risk patients undergoing coronary artery bypass graft and/or cardiac valvular surgery with cardiopulmonary bypass. EP (90 mg/kg) was administered intravenously starting after the induction of general anesthesia followed by 5 more doses of 90 mg/kg administered every 6 h. EP treatment did not reduce major complications within 14 or 28 days of surgery. EP solution has a pH of less than 7, so EP administration may contribute to acidosis, however, no significant safety concerns were discovered during the clinical trial because the adverse event profile for patients receiving EP (*n* = 49) was similar to that of patients receiving placebo (*n* = 53). EP administration has been proved to be safe in a large number of experimental animals, no severe side effect has been reported.

## Conclusions

EP is a novel anti-inflammatory agent and ROS scavenger, this safe and low-cost compound is able to treat multiple inflammatory organ injuries and systemic inflammation in experimental animal models. EP also has a potential to inhibit multiple tumor growth, and this anti-tumor activity may be associated with its anti-inflammatory effect.

## Abbreviations

AKI: Acute kidney injury
ALF: Acute liver failure
ALI: Acute lung injury
APAP: Acetaminophen
ARDS: Acute respiratory distress syndrome
BT: Bacterial translocation
CCI: Cortical contusion injury
DAMP: Damage associated molecular pattern
EP: Ethyl pyruvate
HCC: Hepatocellular carcinoma
HI: Hypoxic-ischemic
HMGB1: High mobility group box 1
ICH: Intracerebral haemorrhage
IDO: Indoleamine 2, 3-dioxygenase
IGF-1: Insulin-like growth factor-1
KCs: Kupffer cells
MAPK: Mitogen activated protein kinase
MODS: Multiple organ dysfunction syndrome
ROS: Reactive oxygen species
SAH: Subarachnoid hemorrhage
SAP: Severe acute pancreatitis
SIRS: Systemic inflammatory response syndrome
TBI: Traumatic brain injury
TCA: The tricarboxylic acid
TLR: Toll like receptor
